# Atrial Remodeling: Evolving Concepts

**Published:** 2003-04-01

**Authors:** Mauro Biffi, Giuseppe Boriani

**Affiliations:** Institute of Cardiology, University of Bologna, Italy

The term "Electrophysiological remodeling" defines the changes of atrial electrophysiologic properties taking place in atrial myocites during atrial fibrillation and/or following periods of sustained atrial fibrillation (AF).

 Early research was prompted by clinical observations: many patients were seen to get through increasingly frequent and longer paroxisms of AF to persistent AF, until chronic AF eventually ensued, without significant changes of underlying heart disease.

Indeed, clinical electrophysiology investigations reported peculiar differences between AF patients and patients without atrial arrhythmias. It was felt that the electrophysiologic milieu was the cause of AF [1,2], until a change of perspective occurred in the early 90's, thanks to experimental research.

In his pioneering work, Wijffels kept goats fibrillating by an implanted device, while measuring atrial refractoriness and its rate adaptation as spontaneous restoration of sinus rhythm occurred. He found that, after several self terminating short bouts of AF, the arrhythmia became considerably longer until a duration of days or weeks was reached. The electrophysiologic companionship of AF pattern transformation consisted of significant shortening of atrial fibrillation cycle, which was paralleled by shortening of atrial ERP and loss of ERP adaptation to heart rate: either flat (no change with shortening cycle length) or inverted (paradoxical shorter ERP at longer cycle length) ERP relationships to cycle length were observed.

This findings had been already observed in AF patients [1,2]; the novelty was that AF itself was the cause of these abnormal electrophysiologic properties, which could in turn promote further recurrences of AF, and arrhythmia persistence in some cases.

By the light of this observations, AF may be seen as self-maintaining by creating a functional electrophysiologic substratum, despite the coexistence of only minor structural heart disease or occasional triggers. This principle has been summarised by Maurits Allessie in the aphorism "AF begets AF".

Following this observations, several studies have addressed the issue of the mechanisms by which atrial electrophysiologic remodeling occurs in different settings: basic electrophysiology, experimental electrophysiology, and clinical electrophysiology. It is nowadays believed that, beyond structural heart disease, changes of the atrial electrophysiologic substratum are responsible for AF recurrence and/or perpetuation.

The aim of this review will be to assess the extent at which atrial remodeling occurs in the experimental animal and in humans, and its relevance to clinical practice.

## Mechanisms of atrial remodeling

Reviewing literature, one has to consider carefully the investigational setting of reported studies, for significant differences exist in electrophysiologic properties and vulnerability to atrial arrhythmias among species (small mammals atria hardly fibrillate, dogs and goats only under pathologic conditions), so that concepts must lean on solid evidence before moving from experimental to clinical electrophysiology, and eventually to clinical practice.

On the other hand, the settings of experimental and basic electrophysiology allow precisely controlled conditions, and a deeper knowledge of the mechanisms, up to the ionic/genomic level.

It is well demonstrated that atrial remodeling is a time-dependent process that develops as an adaptive regulation of cardiac myocites to maintain cell homeostasis against external stressors [3]. The type and extent of remodeling depends on the strength and the duration of exposure to the "stressor": adaptive responses may thus occur at the ionic/genomic level (fully reversible) at short term, or at the cellular level at mid (hibernation, usually reversible) and long term (apoptosis and fibrosis, irreversible).

The most common "stressors" of atrial myocites are: tachycardia (high rate of cell depolarisation), and volume/pressure overload (heart failure syndrome).

The adaptive changes at the ionic level are quite different in the tachycardia-dependent compared to the heart failure setting, as demonstrated by basic electrophysiology, hence we need to focus on different types of "ionic remodeling".

### Tachycardia-dependent remodeling

High atrial rate causes calcium overload. Chronic calcium overload may cause ultrastructural changes that resemble myocardial ischemia or hibernation, and may ultimately lead to irreversible cell damage. To prevent dangerous calcium overload, atrial myocites decrease L-type Ca++ current (ICa) at first by a short-term adaptation (functional inactivation by voltage- and [Ca++] - dependent mechanisms), in the long term by downregulation of ICa [4-7]. Decreased ICa shortens action potential duration (refractoriness) and causes loss of action potential adaptation to heart rate [6,7]: these are the hallmark of tachycardia-dependent remodeling.

The process leading to ICa downregulation implies transcriptional changes at the genomic level, but the signal coupling calcium overload to changes in ion channel expression is unknown. It also remain to be understood whether calcium overload is the only initiating signal in this setting.

The observations by Wijffels et al [8] in the experimental animal are in full agreement with this model of atrial remodeling. In patients with lone paroxysmal AF, Capucci et al [9] demonstrated a strict correlation of atrial refractoriness to AF cycle, and observed progressive shortening of AF cycle during long lasting AF, whereas AF cycle prolonged in self-terminating AF episodes. Acute administration of class 1C drugs was able to prolong AF cycle and terminate long lasting AF episodes [10]. These data are consistent with basic electrophysiology, and are clues that ionic remodeling occurs early during AF, and sets the background for AF recurrence. In the clinical scenario, early AF recurrence after cardioversion of chronic AF is the most threatened complication. In this setting a typically tachycardia-dependent remodeling occurs: in a study of 101 patients [11] we identified loss of atrial refractoriness adaptation to heart rate, short atrial refractoriness and AF duration as the only predictors of AF recurrence at logistic analysis.

In a series of 28 patients, Manios et al [12] similarly observed that failure of action potential adaptation to heart rate was the only prognostic indicator for AF recurrence. Importantly, they observed that the electrophysiological markers of tachycardia-dependent remodeling recover nearly 24 hours after AF cardioversion. This point raises an important question, that is, whether loss of refractoriness adaptation to heart rate is the cause of AF relapse (which could explain only AF relapse within 24-48 hours) or simply a "marker" of a pathologic atrium, which has already undergone some degree of structural remodeling and thus is prone to AF. Up to now, the question is unresolved.

Owing to this knowledge, a considerable amount of work has been done to test the hypothesis that preventing calcium overload by Icablockers would prevent tachycardia-dependent remodeling also. Experimental studies found that pre-treatment with verapamil could attenuate [13] or prevent [14] rapid pacing-induced shortening of atrial refractoriness at short term (< 24 hours of rapid pacing), respectively in goats and dogs. Following these observations, Daoud [15] reported that pre-treatment with verapamil reduced short-duration (5.6 3.9 minutes) AF-induced shortening of atrial refractoriness in humans. In the clinical scenario, Tieleman et al [16] speculated that pre-treatment with verapamil or diltiazem was responsible for reduced AF recurrence in the first month after electrical cardioversion, due to attenuated or rapidly reversed tachycardia-dependent remodeling.

This hypothesis fits poorly with the electrophysiologic findings by Manios [12] who demonstrated that atrial remodeling recovers spontaneously nearly 24 hours after cardioversion: the role of verapamil is indeed unclear. Clinical studies have failed to demonstrated any beneficial effect of verapamil in preventing AF recurrence at long term; indeed, a small effect of verapamil pre-treatment could reasonably be expected only in AF of short duration, and does not affect the electrophysiologic changes occurring with long-lasting AF [17].

Lee et al [17] have performed a very important research in chronically instrumented dogs, in which electrophysiologic data were studied at baseline and after creation of a tachycardia-dependent atrial remodeling, comparing a control group with a verapamil-treated group. Atrial refractoriness, dispersion of atrial refractoriness, rate adaptation of atrial refractoriness, atrial conduction velocity AF inducibility, and AF duration were evaluated respectively after 1 day, 1 week, and 6 weeks of rapid atrial pacing. This study demonstrated that verapamil could only attenuate the refractoriness shortening after 1-day pacing, but could not prevent the electrophysiologic remodeling at long term (1 and 6 weeks). Furthermore, verapamil did not affect atrial inducibility, and increased the duration of induced AF. Consistent with this finding is the observation of increased fragmented activity in paroxysmal AF patients treated by verapamil, as reported by Kumagay [18], which would favour AF persistence. In his study, Lee [17] reported a significant depression of atrial conduction velocity after 6 weeks rapid pacing: this abnormality had no evidence of recovery during two days of follow-up after pacing had been stopped. A similar finding had been previously reported in 2 studies [19,20] of chronic paced dogs, and were related to structural abnormalities occurring along time [21]. All these data make the point that verapamil use is of little value when high atrial rates persist longer than 24 hours, and thus give an explanation to the results observed in clinical practice.

While demonstrating ICa reduction by downregulation of L-type Ca++ channels, Yue et al [6] observed an unchanged inward T-type Ca++ current (ICaT ) even after several weeks of rapid atrial pacing, who could thus represent a continuous "spill" of calcium into atrial cells undergoing high frequency depolarisations. This prompted Fareh et al [22] to investigate the effect of mibefradil, a pure T-type Ca++ blocker, in a chronic rapid-pacing dog model to prevent the development of an electrophysiologic substrate for AF. After 1 week of rapid pacing, mibefradil-treated dogs did not differ from control dogs (no rapid pacing) in any electrophysiologic parameter, except for a slightly increased heterogeneity of atrial refractoriness, whereas placebo-treated dogs had developed all the characteristic abnormalities of tachycardia-dependent remodeling (easy AF inducibility included). A potential direct electrophysiologic effect of mibefradil was also excluded in this study, thus supporting the evidence that prevention of tachycardia-induced remodeling as the only protective effect of mibefradil. Indeed, the AF-promoting effects of rapid pacing were negatively correlated to mibefradil plasma concentrations, showing a dose-dependent protection against atrial remodeling.

In a similar study on dogs, Fareh et al [23] also demonstrated that prevention of tachycardia-dependent remodeling is possible with the T-type Ca++ channel blocker mibefradil, but not with L-type Ca++ channel blockers.

There is considerable evidence that, in tachycardia-dependent remodeling, calcium overload plays an important role either at short or at long term if high atrial rates persist. In the clinical setting, small benefits may be observed at short term by verapamil, whereas T-type Ca++ channel blockers seem promising to prevent long term remodeling during persistent AF.

These data support the concept that new therapies should target the development of the substrate for AF, as opposed to "traditional" antiarrhythmic therapy which aims to modify the product of the arrhythmic substrate.

### Heart failure-dependent remodeling

Heart failure (HF) is the most important clinical syndrome associated to AF [24]. Heart failure causes important ionic remodeling either at the atrial or at the ventricular level by powerful neurohormonal changes. The study of atrial remodeling in HF is very difficult in humans because of the structural changes caused by underlying myocardial pathology, and because of the confounding effect of drugs acting on the neurohormonal milieu and of antiarrhythmic agents. For this reason most of available data rely upon basic and experimental research, most often on ventricular myocites.

Heavy alterations in calcium handling are prominent in ventricular myocites: the sarcoplasmic carrier of Ca++ is downregulated in failing myocites [25], leading to increased cytosolic [Ca++] as a maladaptive attempt to maintain contractility. Sodium-calcium exchanger current (NCX) is instead upregulated in HF, acting to compensate the impaired Ca++ removal due to the sarcoplasmic carrier [26-28], to protect contractile function and cellular Ca++ homeostasis. NCX exchanges one Ca++ for three Na+ , thus carrying a net current in the direction of Na+ transport. The net current carried by NCX may cause delayed afterdepolarisation (DAD) in certain electrophysiologic situations, which proved to be arrhythmogenic in HF [29].

Danshi li et al. [30] created a canine model of rapid ventricular pacing-induced HF, and observed for the first time in atrial myocites a decrease in ICa, in Na+ current (Ito), in slow rectifying K+ current (Iks), and an increase in NCX. Heart failure decreased both Iks and ICa 30%, whereas atrial tachycardia decreases ICa 70% without affecting Ik [6]. The electrophysiologic manifestations were an unchanged action potential duration (APD) at slow heart rates compared to control dogs, whereas an increase of APD at faster high rates was observed.

Thus, a peculiar type of remodeling with an attenuated rate-adaptation of atrial refractoriness and long APD at high rates is observed in experimental HF. This is quite different from atrial tachycardia-dependent remodeling, which shows decreased APD at any cycle length and flat or reversed adaptation of refractoriness to heart rate.

The mechanisms of AF onset in the setting of HF must be very different. Structural changes (interstitial fibrosis) related to underlying disease cause conduction abnormalities that predispose to reentrant arrhythmias, increased NCX may promote DADs by transient inward depolarising current, which may be enhanced by high cathecolamine levels and by decreased Iks. It seems then likely that AF may be initiated by triggered activity, in the HF setting.

If a different sort of atrial remodeling occurs in HF, peculiar interventions for targeting this arrhythmogenic substrate should be sought.

Danshi Li and co-workers [31] performed an elegant investigation on 15 dogs with HF induced by rapid ventricular pacing compared to 10 dogs without HF subjected to rapid atrial pacing (RAP), evaluating the effect of dofetilide on vulnerability to AF. It was observed that dofetilide, a pure Ikr blocker, was extremely effective in terminating AF and in preventing AF reinduction in dogs with HF-dependent AF. On the contrary, dofetilide was completely ineffective in dogs with atrial tachycardia-dependent AF. The electrophysiologic findings were striking: atrial refractoriness was shorter and showed less rate adaptation in RAP vs HF dogs; dofetilide increased refractoriness significantly and to a greater extent in HF dogs, whereas it had nonsignificant effects in RAP dogs.

This finding was related to the increased dependency of atrial refractoriness on Ikr in HF dogs, due to downregulation of Iks occurring in HF dogs.

Significant clinical results were indeed observed by Torp-Pedersen et al [32] investigating dofetilide effects in advanced HF patients (class III-IV): a significant proportion of AF patient converted to sinus rhythm by dofetilide, and dofetilide patients had a significantly higher proportion of sinus rhythm persistence during clinical follow up compared to control patients.

Nonetheless, AF incidence increases with the severity and the duration of HF despite therapeutic interventions.

Shinigawa et al [33] studied the electrophysiologic remodeling occurring during atrial tachycardia in a canine model when HF was superimposed, and also after reversal of HF was allowed to take place. They observed that the electrophysiologic changes related to atrial tachycardia were completely attenuated by HF, and that reversal of HF led to normalisation of both ventricular and atrial volume and contractility, with recovery of atrial electrophysiologic abnormalities. However, extensive interstitial fibrosis had developed compared to a group of control dogs, and this invariably caused local conduction slowing and heterogeneity of conduction velocity. This changes were irreversible.

Interestingly, in the same experimental canine model of HF, Danshi Li et al [34] reported that angiotensin II causes structural atrial remodeling inducing collagen synthesis by fibroblasts, and that enalapril interferes with signal transduction at the cellular level, thereby reducing the amount of interstitial fibrosis by 30% compared to control dogs or to hydralazine-treated dogs.

These two studies carry the important notion of targeting the development of the arrhythmogenic substrate understanding the pathophysiologic mechanisms of the disease or of the clinical syndrome.

It is interesting to note that, either in atrial tachycardia-dependent or in HF-dependent remodeling, both reversible and irreversible changes occur. In either situation, the abnormalities due to ionic remodeling (APD, refractoriness and its adaptation to heart rate) appear to be reversible when the "stressor" is removed, but in the same time structural changes at the cellular level have occurred, which are irreversible. This "structural remodeling" adds slowing and heterogeneity of conduction velocity to the abnormalities of refractoriness in both situations, and may explain the reduced efficacy of medical interventions in patients with persistent or permanent AF.

Experimental research has clearly demonstrated that "electrophysiologically" different arrhythmias share a common electrocardiographic picture of atrial fibrillation, and that the underlying differences imply peculiar therapeutic interventions.

All these investigations have brought in the clinical arena a new way of thinking antiarrhythmic therapy: development of new drugs should be guided by the principle of targeting the specific ionic currents depending on the pathophysiologic mechanisms of the arrhythmia, to achieve the optimal ratio between efficacy and safety. Also, the concept of preventing structural remodeling of atrial myocites should be investigated and promptly brought into clinical practice.
